# Prediction models constructed for Hashimoto’s thyroiditis risk based on clinical and laboratory factors

**DOI:** 10.3389/fendo.2022.886953

**Published:** 2022-08-08

**Authors:** Peng Li, Fang Liu, Minsu Zhao, Shaokai Xu, Ping Li, Jingang Cao, Dongming Tian, Yaopeng Tan, Lina Zheng, Xia Cao, Yingxia Pan, Hui Tang, Yuanyuan Wu, Yi Sun

**Affiliations:** ^1^ Department of Breast Surgery, Xuchang Central Hospital, Xuchang, China; ^2^ Health Management Center, Kaifeng Central Hospital, Kaifeng, China; ^3^ Department of Endocrinology, Jincheng People’s Hospital, Jincheng City, China; ^4^ Department of Medicine, Shanghai Biotecan Pharmaceuticals Co., Ltd., Shanghai, China; ^5^ Shanghai Zhangjiang Institute of Medical Innovation, Shanghai, China

**Keywords:** Hashimoto’s thyroiditis, thyroid cancer, precise diagnosis, machine learning modeling, risk factors

## Abstract

**Background:**

Hashimoto’s thyroiditis (HT) frequently occurs among autoimmune diseases and may simultaneously appear with thyroid cancer. However, it is difficult to diagnose HT at an early stage just by clinical symptoms. Thus, it is urgent to integrate multiple clinical and laboratory factors for the early diagnosis and risk prediction of HT.

**Methods:**

We recruited 1,303 participants, including 866 non-HT controls and 437 diagnosed HT patients. 44 HT patients also had thyroid cancer. Firstly, we compared the difference in thyroid goiter degrees between controls and patients. Secondly, we collected 15 factors and analyzed their significant differences between controls and HT patients, including age, body mass index, gender, history of diabetes, degrees of thyroid goiter, UIC, 25-(OH)D, FT3, FT4, TSH, TAG, TC, FPG, low-density lipoprotein cholesterol, and high-density lipoprotein cholesterol. Thirdly, logistic regression analysis demonstrated the risk factors for HT. For machine learning modeling of HT and thyroid cancer, we conducted the establishment and evaluation of six models in training and test sets.

**Results:**

The degrees of thyroid goiter were significantly different among controls, HT patients without cancer (HT-C), and HT patients with thyroid cancer (HT+C). Most factors had significant differences between controls and patients. Logistic regression analysis confirmed diabetes, UIC, FT3, and TSH as important risk factors for HT. The AUC scores of XGBoost, LR, SVM, and MLP models indicated appropriate predictive power for HT. The features were arranged by their importance, among which, 25-(OH)D, FT4, and TSH were the top three high-ranking factors.

**Conclusions:**

We firstly analyzed comprehensive factors of HT patients. The proposed machine learning modeling, combined with multiple factors, are efficient for thyroid diagnosis. These discoveries will extensively promote precise diagnosis, personalized therapies, and reduce unnecessary cost for thyroid diseases.

## Introduction

Hashimoto’s thyroiditis (HT) is a chronic disease of autoimmune thyroid disorder (AITD) with high incidence, which is a common cause of hypothyroidism. It is mainly manifested by thyroid lymphocytic infiltration and fibrosis, as well as positive thyroid-specific antibodies in peripheral blood (1). Targeting the imaging diagnosis of the thyroid, the Thyroid Imaging Reporting and Data System (TIRADS) was established in many countries (2–4), including China (5). The best imaging method for diagnosing thyroid diseases is ultrasonography which provides precise dimensions of thyroid goiters (6). HT is the most frequent cause of goiters which are initially firm, bumpy, symmetric, and painless (7). However, HT is usually not recognized and diagnosed in the early stage and therefore, HT is more likely to deteriorate into papillary thyroid carcinoma (PTC) than nodular goiter (8, 9). Thus, it is necessary to establish prediction models for HT diagnosis at an early stage.

According to numerous reports, the development of thyroid diseases, especially thyroid cancer, is correlated with many risk factors, among which the main factors may optimize diagnostic techniques and treatment methods. For instance, iodine is an essential raw material for the synthesis of thyroid hormones (10). Studies have shown that the harm of iodine deficiency to individuals is significantly more serious than the harm caused by excessive iodine consumption (11). Insufficient iodine intake can cause elevated thyroid stimulating hormone (TSH) levels, leading to clinical hypothyroidism by the regulation through intact hypothalamus-pituitary-thyroid axis (12, 13). The long-term iodine deficiency can lead to HT and even thyroid cancer. However, thyroid cancer is seemingly more prevalent in areas with excessive iodine intake which will induce the reduction of thyroid hormone synthesis. Susceptible population with excessive iodine intake may either induce hypothyroidism, such as HT, or cause hyperthyroidism, such as Grave’s disease (GD) (14, 15), which suggests a complex relationship between iodine levels with AITD (16). The iodine uptakes of HT patients are usually low and can be tested by urinary iodine concentration (UIC) (17). Moreover, vitamin D is usually lacking in patients suffering from HT (18). It is reported that the risk of developing HT and thyroid cancer will increase with low 25-hydroxy vitamin D (25-(OH)D) levels, which can be improved by the supplementation of vitamin D (19).

In addition to malnutrition, the high levels of antibodies against thyroid antigens have been positively correlated with the clinical diagnosis of HT, especially for the thyroglobulin antibody (TGAb) against thyroid peroxidase (TPO) and the thyroid peroxidase antibody (TPOAb) against thyroglobulin (TG) (20–22). Also, the proper levels of inherent hormone metabolism, such as free thyroxine (FT4) and free triiodothyronine (FT3), along with fatty acid intake play important roles in the course of HT and thyroid cancer (19, 23). It is reported that other endocrine diseases, such as diabetes and hyperlipemia, are often concurrent in HT patients whose fasting plasma glucose (FPG) may be disordered (24, 25), while triglyceride (TAG), total cholesterol (TC) and low-density lipoprotein cholesterol (LDL-C) may be significantly lower than in healthy people (26).

In recent years, the applications of novel computer-aided diagnosis based on bioinformatic engineering has increasingly played appealing roles in the clinical diagnoses of multiple disorders. Many research studies on computer-aided diagnoses of disorders, including thyroid diseases, have been carried out. It is reported that computer-aided diagnosis can offer efficient dataset processing, accurate diagnosis, clinical decision-making support, and even potential treatment options for thyroid diagnosis (27–30). Computer-aided diagnosis of HT has been carried out since 2009 (31). However, current computer-aided studies basically focus on thyroid imaging and only a few research studies involve molecular levels. According to a 2019 study, thyroid antibodies and hormones were involved in establishing a machine learning model for the quantitative and convenient diagnosis of HT distinguished from the Graves’ disease (32), but these factors were not comprehensive enough. Recent studies on HT found that mathematical modeling highlighted T helper lymphocytes TH1, TH17, and the bacterial balance of the gut microbiota activities as risk factors for HT development (33).

In this study, we collected fifteen clinical characteristics and laboratory data to study their significant correlations with HT and thyroid cancer. Furthermore, we established machine learning models of HT development and thyroid cancer based on twelve factors. The study may offer reference in the diagnosis and prevention of HT, which also effectively reduces the economic burden on patients and save limited medical resources.

## Methods

### Recruitment and examination of participants

From July 2018 to January 2021, total 1,344 participants were recruited from Xuchang Central Hospital, Kaifeng Central Hospital, and Jincheng People’s Hospital. This total included 885 non-HT controls and 459 diagnosed HT patients. All participants signed informed consents. The exclusion criteria in this study were incomplete clinical information, controls with thyroid cancer, and abnormal values. After screening, the cases of controls and HT patients were 866 and 437, respectively. The total factors were collected as follows: age, body mass index (BMI), gender, history of diabetes, degrees of thyroid goiter, thyroid cancer, UIC, 25-(OH)D, FT3, FT4, TSH, TAG, TC, FPG, LDL-C, and high-density lipoprotein cholesterol (HDL-C).

### Inclusion criteria of HT patients and controls

The clinical criteria of HT are diffuse thyroid goiter, tough tissue, hypoechoic, disordered echoes on the thyroid, nodular changes with regular borders diagnosed by B-mode ultrasound imaging, along with increased TPOAb and/or TGAb in sera (34–36). The controls had none or merely part of those changes in the thyroid gland. By means of clinical inspection and palpation, thyroid goiter was divided into three degrees (I, II, III) according to their sizes (Degree I: touchable but invisible; Degree II: touchable and visible, limited to posterior margin of sternocleidomastoid muscle; Degree III: touchable and visible, greater than posterior margin of sternocleidomastoid muscle). After data screening, 1,303 non-HT controls and HT patients were enrolled in the cohort from the initial 1,344 participants.

### Ultrasound examination of thyroid glands

Ultrasound examinations were performed and pictured with the Vinno E30 scanner (Vinno Technology, Jiangsu, China). The frequency bandwidth of broadband linear array transducer (X6-16L) is 10 ~ 14 MHz. In comparison with the controls, ultrasound features of HT patients were diffusely hypoechoic nodules on thyroid glands, visible sheet-like hypoechoic areas, and fibrous hyperechoic areas (**Additional file 1**). Ultrasound features of PTC differentiated from benign goiter, including irregular and rough borders, aspect ratios of nodules, microcalcification, increased blood flow, disordered and hypoechoic appearance on fibrous hyperplasia (37).

### Microscopic examination of cytopathological sections

Microscopic examinations were only available for HT patients who potentially had thyroid cancer. The specimens of patients’ thyroid glands were taken from biopsy or surgery and made into intraoperative cytology smears. The histopathological specimens were fixed in formalin solution and observed by Hematoxylin-Eosin staining. By means of microscope (Leica, DM 2000) examination, the postoperative diagnostic results were classified into normal, HT-type inflamed, and cancerous cells by their cytological appearance (**Additional file 2**). The pathological photographs were taken under magnification of 200. The pathological features of specimens taken from HT patients are thyroid follicles with Hürthle-cell metaplasia, lymphoid follicles with germinal centers, minimal colloid in follicles, and polymorphic lymphoid cells (38). The typical cytological recognition of PTC is an extensive papillary pattern, lymphoplasmacytic infiltrate, follicular variant, tall cell variant, and psammoma bodies. The typical nuclear characteristics of PTC are ground-glass nuclei, nuclear grooves, and round intranuclear pseudoinclusions (39, 40).

### Collection and detection of urine and blood samples

Urine and blood samples were collected and detected from all participants at the time of initial diagnosis. The UIC in urine samples were detected by inductively coupled plasma-mass spectrometry (ICP-MS) (Agilent Technologies, Inc., Tokyo, Japan) (41). The vitamin D detection includes vitamin D3 (cholecalciferol) and vitamin D2 (ergocalciferol) levels, which were measured by 25-(OH)D in serum. The 25-(OH)D levels were measured using Ultrahigh Performance Liquid Chromatography-Tandem Mass Spectrometry (UPLC-MS/MS; UPLC-MS/MS ACQUITY UPLC I-class Xevo TQD, Waters Corporation) on the basis of the manufacturer’s protocol. Serum was separated from fasting blood samples by spinning at 3,000 rpm for 15 min in 4°C. The laboratory data of 25-(OH)D, FT3, FT4, TSH, FPG, and lipid profiles were detected using an automatic biochemical analyzer (Beckman Coulter, Inc., CA, USA). TPOAb, TGAb and TG were detected using an automatic biochemical analyzer (Roche Ltd., Switzerland). All the laboratory data were measured in serum, except for UIC. All the factors mentioned above are summarized in **Table 1**.

**Table 1:** Clinical characteristics of controls and HT patients.

Character	Control (n=866)	HT-C (n=393)	HT+C (n=44)	*P*-value
Goiter degree				<0.0001
None	859 (99.19%)	36 (9.16%) ↓	1 (2.27%) ↓	
I	3 (0.35%)	183 (46.56%) ↑	13 (29.55%) ↑	
II	2 (0.23%)	165 (41.98%) ↑	29 (65.91%) ↑	
III	2 (0.23%)	9 (2.29%) ↑	1 (2.27%) ↑	
Age (years)	54.06 ± 13.50	47.66 ± 13.09 ↓	51.45 ± 11.86 ↓	<0.0001
BMI (kg/m^2^)	24.49 ± 3.31	23.15 ± 3.55 ↓	24.64 ± 3.31 ↑	<0.0001
Gender				<0.0001
Male	508 (58. 66%)	49 (12.47%) ↓	8 (18.18%) ↓	
Female	358 (41.34%)	344 (87.53%) ↑	36 (81.82%) ↑	
Diabetes				0.0433
Yes	28 (3.23%)	24 (6.11%) ↑	3 (6.82%) ↑	
No	838 (96.77%)	369 (93.89%) ↓	41 (93.18%) ↓	

The table shows the statistics of clinical characteristics and laboratory results of controls, HT patients and patients with thyroid cancer. There were significant differences in most factors between controls and HT patients. BMI, body mass index. P-values were calculated by Kruskal–Wallis H test or Chi-square test among the triple groups. Symbols "↓ and ↑" represent “reduced and increased” change in mean values compared to corresponding controls.

### Machine learning modeling of HT

A total of 275 controls and 361 HT patients were recruited to construct the HT machine learning model with Python Software (version 3.8). Multiple factors were selected including gender, age, BMI, diabetes, 25-(OH)D, UIC, FT3, FT4, TSH, FPG, TC, and TAG.

Six machine learning models, including K-nearest neighbor classifier (KNN), logistic regression (LR), support vector machine (SVM), decision tree model (DT), multilayer perceptron network (MLP), and eXtreme Gradient Boosting (XGBoost) models were utilized. StratifiedKFold (k= 5) was used to divide samples into the training set and the test set. After the training, the six models were evaluated in the test set. The accuracy, precision, recall (sensitivity), F1 scores, and area under curve (AUC) scores of each model were calculated, respectively. The selected features were ranked in sequence of their importance for the XGBoost model. The receiver operating characteristic curve (ROC) of each model displayed the judgment ability of the model.

### Statistical analysis

Statistical analyses were performed using SPSS 27.0 and Graphpad Prism 8.0 software. Significant difference analysis of unordered variables and rank variables in distribution between independent groups were separately assessed with the Chi-square test and the Ridit test. Significant difference analysis of continuous variables between the two groups were separately compared by the Mann–Whitney U test for large sample sizes and the Kolmogorov-Smirnov test for small sample sizes. Significant difference analysis of continuous variables among triple groups were compared by the Kruskal–Wallis H test. The quantitative data were displayed as mean ± standard deviation.

Binary logistic regression analysis was performed to investigate the risk factors for HT development by SPSS software with the Hosmer-Lemeshow test. The odds ratio (OR) corresponded to the correlation between factors and HT development. OR > 1 represented the risk factor triggering HT, while OR < 1 represented the protective factor restricting HT. The analysis results were adjusted for each factor and considered statistically significant when *P*-values of tests were less than 0.05.

## Results

### Cohorts of participants

**Figure 1:**
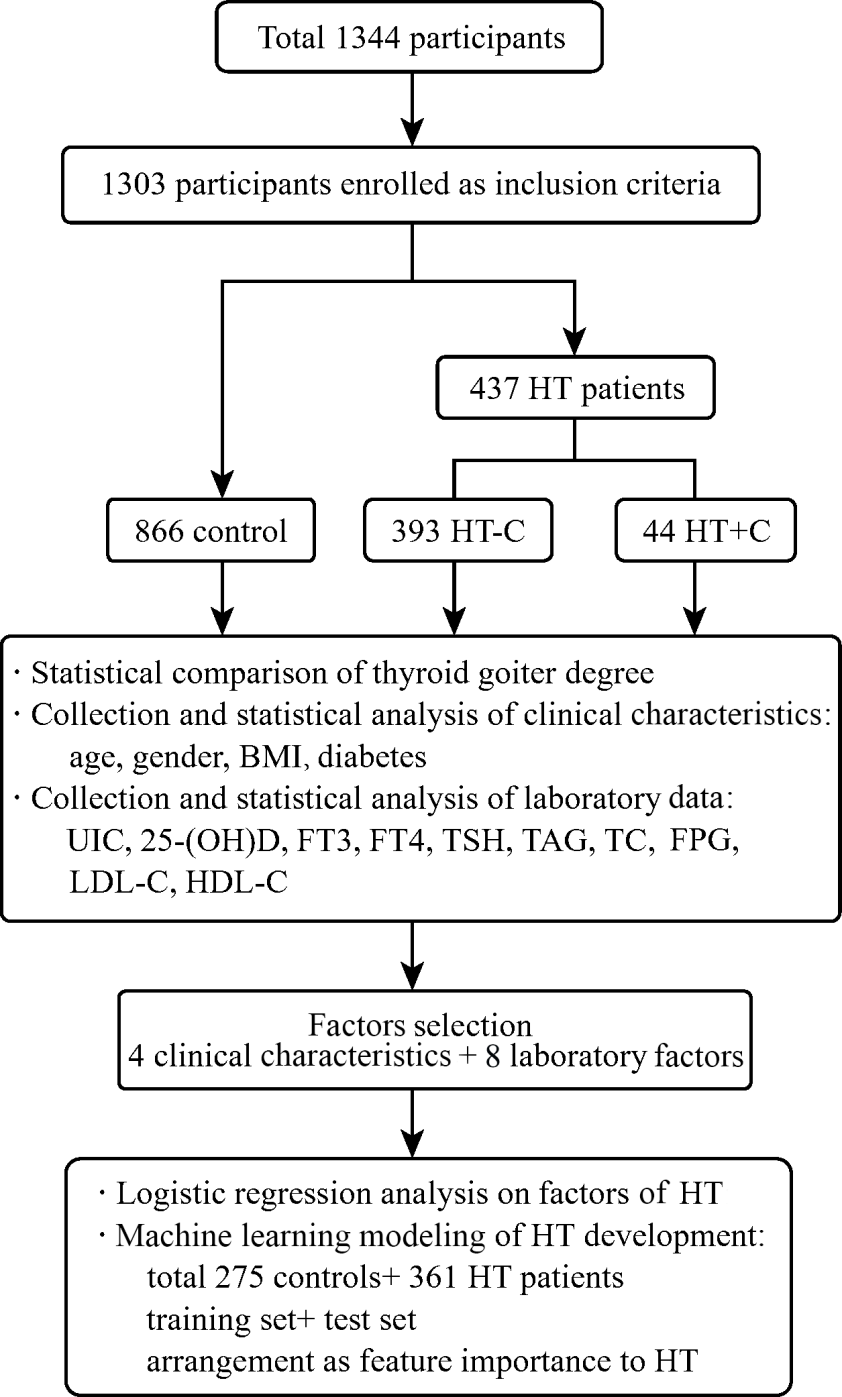
Overview of the cohort study. The flow chart shows the participants selection and classification, statistical analysis and logistic regression analysis on multiple factors of controls and patients, as well as machine learning modeling and evaluation of HT development.

Among total 1,344 participants, 1,303 non-HT controls and first-visit HT patients were enrolled after screening. According to ultrasound imaging and cytopathological examinations, they were divided into 866 controls and 437 patients. The 437 diagnosed HT patients were divided into 393 HT patients without cancer (HT-C) and 44 HT patients with thyroid cancer (HT+C). Then, multiple clinical characteristics and laboratory test data were collected as shown in **Figure 1**.

### Clinical characteristics of participants

Compared to controls, the significant differences in goiter degrees between HT-C and HT+C patients are shown in **Table 1**. It indicates that 357 HT-C patients (90.83%) and 43 HT+C patients (97.73%) developed goiter while only 7 controls (0.81%) had goiter. Specifically, Degree I of goiter mostly appeared in 183 HT-C patients (46.56%), Degree II of goiter mostly appeared in 29 HT+C patients (65.91%), Degree III of goiter mostly appeared in 9 HT-C patients (2.29%). In contrast, Degrees I to III of goiter among controls were 3, 2, and 2, which all accounted for the least (0.35%, 0.23%, and 0.23%, respectively). Therefore, the proportions of goiter degrees were significantly different between the HT-C and HT+C patients compared to the controls.

Among HT patients, 344 of 393 (87.53%) HT-C and 36 of 44 (81.82%) HT+C patients were women while 358 of 866 (41.34%) controls were women. 24 of 393 (6.11%) HT-C and 3 of 44 (6.82%) HT+C patients were incorporated with diabetes while 28 of 866 (3.23%) controls had diabetes. Statistics on significant tests indicated that women (*P* < 0.0001) and diabetic patients (*P* = 0.0433) were more likely to develop HT disease. Besides, compared to controls, the clinical characteristics, including age, BMI, gender, and diabetes all played significant roles in HT-C patients. However, only BMI had a statistical difference between HT-C and HT+C patients. The average BMI of HT-C was significantly lower than those in controls and HT+C patients. Significant difference in continuous variables were compared by the Mann–Whitney U test between the two groups and the Kruskal–Wallis H test among the three groups. Significant difference in categorical variables were compared by the Chi-square test.

### Laboratory test data of participants

**Figure 2:**
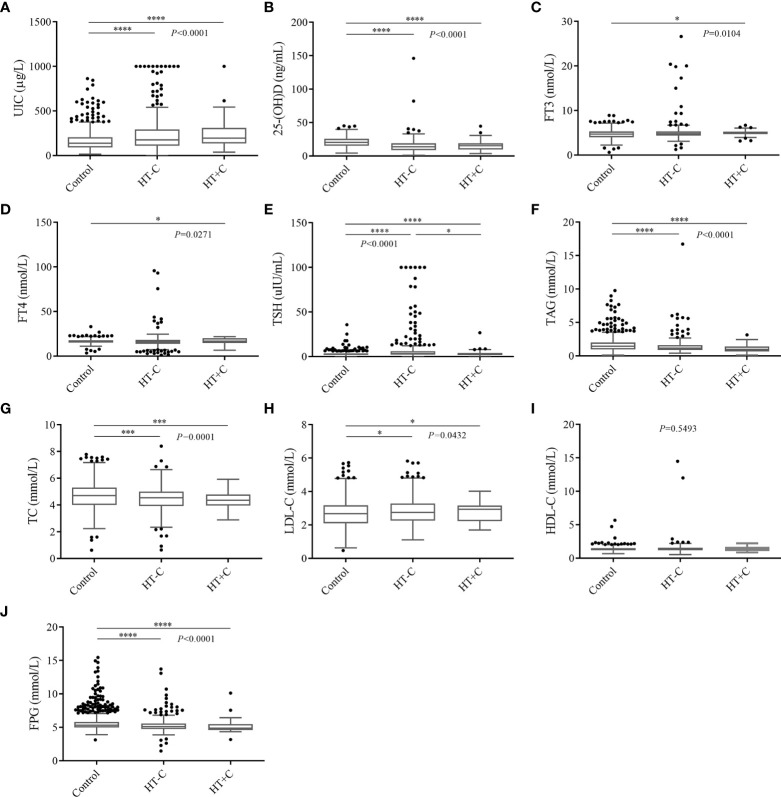
Multiple comparative analysis testing of laboratory data among controls and HT patients. **(A)** UIC, **(B)** 25-(OH)D, **(C)** FT3, **(D)** FT4, **(E)** TSH, **(F)** TAG, **(G)** TC, **(H)** LDL-C, **(I)** HDL-C, **(J)** FPG. *P < 0.05, ***P < 0.001, ****P < 0.0001. UIC, urinary iodine concentrations; 25-(OH)D, 25 hydroxyvitamin D; FT3, free triiodothyronine; FT4, free thyroxine; TSH, thyroid stimulating hormone; TAG, triglyceride; TC, total cholesterol; LDL-C, low density lipoprotein cholesterol; HDL-C, high density lipoprotein cholesterol; FPG, fasting plasma glucose.

Compared to controls, the 437 HT patients significantly had advantageous distributions in averages of the following factors: UIC (162.13 ± 110.58) μg/L vs. (243.99 ± 203.27) μg/L, FT3 (4.71 ± 1.01) nmol/L vs. (5.04 ± 2.01) nmol/L, TSH (2.86 ± 2.31) uIU/mL vs. (7.07 ± 14.69) uIU/mL, LDL-C (2.67 ± 0.80) mmol/L vs. (2.81 ± 0.82) mmol/L, separately. On the contrary, the significant inferior factors in HT patients were as follows: age (54.06 ± 13.50) years vs. (48.04 ± 13.01) years, BMI (24.49 ± 3.31) kg/m^2^ vs. (23.30 ± 3.55) kg/m^2^, 25-(OH)D (20.72 ± 7.35) ng/mL vs. (15.03 ± 10.37) ng/mL, FT4 (16.41 ± 2.35) nmol/L vs. (16.36 ± 7.71) nmol/L, TAG (1.66 ± 1.09) mmol/L vs. (1.36 ± 1.13) mmol/L, TC (4.70 ± 0.99) mmol/L vs. (4.44 ± 0.89) mmol/L and FPG (5.66 ± 1.34) mmol/L vs. (5.27 ± 1.13) mmol/L. However, the HDL-C level of (1.35 ± 0.33) mmol/L in controls had no statistical significance with (1.44 ± 0.97) mmol/L in HT patients. Among laboratory data of HT-C patients, UIC, TSH, and LDL-C are significantly higher while 25-(OH)D, TAG, TC, and FPG are significantly lower than those of the controls. Nevertheless, only TSH was significant different compared to HT+C patients (**Figure 2**). In conclusion, apart from HDL-C, most clinical characteristics indeed have diagnostic values for HT while they have no indicative meaning for thyroid cancer.

### Logistic regression analysis on risk factors for HT development

The selected factors collected from 275 controls and 361 HT patients were independently analyzed for the HT risk factors with binary logistic regression. The OR corresponded to the probability that factors may promote HT (OR > 1) or restrict HT development (OR < 1). The adjusted result shows that diabetes (OR = 6.617, *P* < 0.001), UIC (OR = 1.001, *P* = 0.005), FT3 (OR = 1.721, *P* < 0.001), and TSH (OR = 1.128, *P* < 0.001) significantly have risk effects on HT development. In contrast, gender (OR = 0.264, *P* < 0.001), 25-(OH)D (OR = 0.945, *P* < 0.001) and FPG (OR = 0.664, *P* < 0.001) may protect HT development. Thus, diabetes, UIC, FT3, and TSH can be identified as important risk factors for HT development (**Table 2**).

**Table 2:** Logistic regression analysis on risk factors for HT.

Characters	Wald	*P*-value	OR	95% CI for OR	
				Lower	Upper
Gender	33.053	<0.001	0.264	0.167	0.415
Diabetes	12.962	<0.001	6.617	2.365	18.512
UIC	7.804	0.005	1.001	1.000	1.003
25-(OH)D	19.761	<0.001	0.945	0.922	0.969
FT3	15.929	<0.001	1.721	1.318	2.246
TSH	11.203	<0.001	1.128	1.051	1.211
FPG	12.804	<0.001	0.664	0.531	0.831

The adjusted quantification of relative risk relations between factors and HT. OR, odds ratio; UIC, urinary iodine concentrations; 25-(OH)D, 25 hydroxyvitamin D; FT3, free triiodothyronine; TSH, thyroid stimulating hormone; FPG, fasting plasma glucose.

### Machine learning modeling of HT and thyroid cancer development

**Figure 3:**
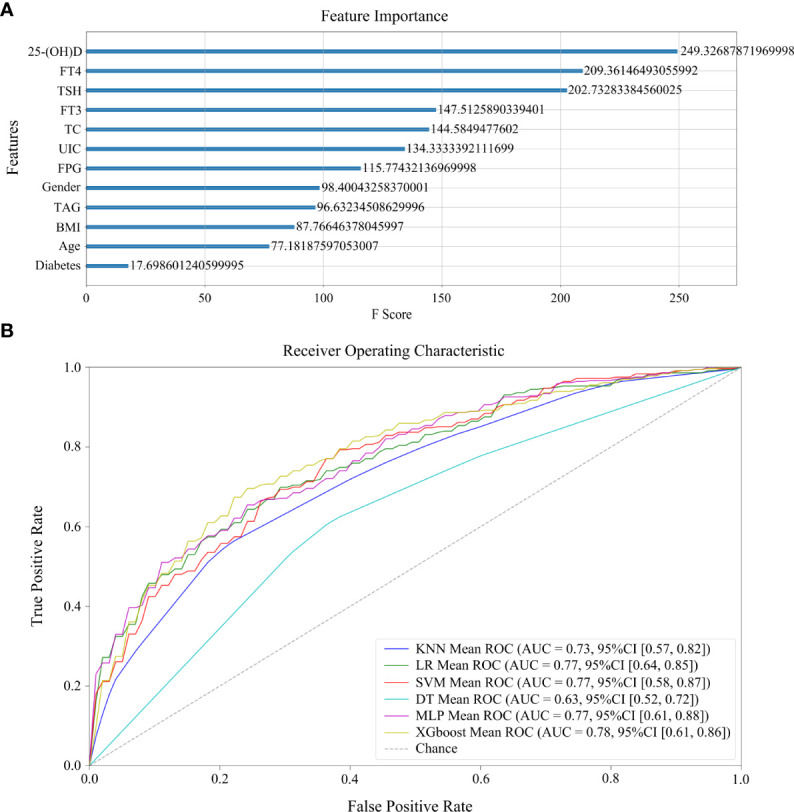
Machine learning models of HT development. **(A)** The rank of feature importance in model construction, including clinical and laboratory factors, as well as their corresponding F scores. **(B)** The ROC curves of six models.

A total 275 controls and 361 HT patients with complete clinical characteristics and laboratory results were selected for modeling. Six models were established by different machine learning algorithms. The ROC curves of six models are displayed in **Figure 3B**, among which the XGBoost model has the highest AUC score (0.781673). Also, the AUC scores of LR (0.767496), SVM (0.766069), and MLP (0.775333) models were superior. However, the AUC scores of KNN and DT models were lower than 0.75, which were not practicable for HT prediction. The performance of six machine learning models verifies that the XGBoost model is the best in accuracy (0.729774) and precision (0.770717) (**Table 3**). The following features were arranged according to their importance for the XGboost model, including 25-(OH)D, FT4, TSH, FT3, TC, UIC, FPG, gender, TAG, BMI, age, and diabetes (**Figure 3A**). Among all features, 25-(OH)D played the most important role in HT risk model construction.

**Table 3:** Performance summary of six machine learning models of HT.

Models	Accuracy	Precision	Recall	F1 Score	AUC Score
KNN	0.681029	0.746247	0.681507	0.710569	0.728307
LR	0.701378	0.730701	0.780822	0.749290	0.767496
SVM	0.710839	0.720756	0.825304	0.766319	0.766069
DT	0.643135	0.680359	0.706202	0.691899	0.633101
MLP	0.721863	0.746918	0.797603	0.767313	0.775333
XGBoost	0.729774	0.770717	0.772755	0.767228	0.781673

AUC, area under curve; KNN, k-nearest neighbor classifier; LR, logistic regression; SVM, a support vector machine; DT, the decision tree model; MLP, the multilayer perceptron network; XGBoost, eXtreme Gradient Boosting.

For the purpose of further studying whether these risk factors are also available for thyroid cancer developing from HT, machine learning models were established between 318 HT-C patients and 43 HT+C patients using the similar methods above. However, the AUC score of the most optimal model in the test set was lower than 0.65, which indicated that these factors are not suitable for machine learning of thyroid malignancy in our cohort (**Additional file 3**).

## Discussion

Presently, HT is the most common chronic autoimmune disease worldwide (42), which has the risk of occurring with thyroid cancer or deteriorating into thyroid cancer, such as PTC (43–45). Apart from thyroid cancer, HT may also lead to other disorders (46, 47). However, some difficulties still exist in the diagnosis of HT. For example, ultrasonography is an essential and prevalent noninvasive tool for diagnosis of HT (48), but the correct diagnosis of HT is based on the extensive clinical experience of physicians. Besides, it is difficult for ultrasonography to distinguish HT from other diseases when HT coexists with other thyroid disorders (49). Conversely, the diagnosis of HT based on an invasive cytological examination of ultrasound-guided fine-needle biopsy or surgical samples should also be optimized (49), since the examination process needs at least three days and the recognition of marked cells needs more time as well as specialized knowledge of cytology. Thus, it is urgent to develop new precise diagnostic methods for early-stage HT.

In our study, the cases of thyroid goiter accounted for 0.81%, 90.83%, and 97.73% of the 866 non-HT controls, 357 HT-C patients, and 43 HT+C patients, respectively. Besides, 44 (9.59%) out of 49 HT patients also had thyroid cancer. Thus, the thyroid goiter significantly has a positive relationship with HT development. The incidence of HT is clinically insidious and sometimes asymptomatic and it has been reported that a number of HT patients (38.80%) were significantly asymptomatic (50). If the thyroid goiter is not adequately investigated or patients are asymptomatic, it is likely to remain undiagnosed though being in an early phase of HT. Therefore, correctly distinguishing benign and malignant thyroid goiters is important for the treatment and prognosis assessment of HT.

There were multiple factors that significantly changed between HT patients and controls in our study. Firstly, the levels of TSH elevated likewise in HT patients compared to controls. The FT4 level significantly reduced while the FT3 level significantly elevated in HT patients compared to controls. In order to diagnose HT more conveniently and quickly, it is preferred to simultaneously inspect the thyroid functions, hormones, and antibodies in the blood, including TSH, FT3, and FT4. It was reported that in the HT patients, there may be a clinical manifestation of elevated TSH, along with poor FT3 and FT4 levels (12, 13, 19, 23, 32). Secondly, there has been much research on the physiological changes in the blood and urine of HT patients (18, 51, 52). For the HT patients we studied, the average 25-(OH)D level was significantly reduced while UIC was significantly elevated compared to controls. Micronutrients, such as iodine and vitamin D, are often found to be deficient in HT patients (17, 53). In our study, the levels of FPG, TAG, and TC were all significantly decreased while LDL-C was significantly increased in HT patients compared to controls. Blood glucose and blood lipid, including FPG, TAG, TC, and LDL-C are indeed found to be decreased in HT patients (24–26). Thirdly, except for laboratory results, many clinical characteristics are also associated with HT development. In our study, we found that gender, age, and BMI were significantly correlated with HT. Diabetes also had a significant positive correlation with HT. According to previous studies, middle-aged women are more likely to suffer from HT than men (32). In general, the average BMI of HT patients tends to be lower than controls (53). Also, patients with an endocrine disorder, such as diabetes, also have a potential HT risk (25). However, many other autoimmune disorders are associated to HT, such as rheumatic disorders, autoimmune hemolytic anemia, and immune thrombocytopenic purpura, which may become potential risk factors for HT development (54–57).

As for thyroid cancer, in our study, only BMI and TSH had significantly positive impacts on the development of thyroid cancer compared to controls. Most thyroid cancer patients had the same altered tendency as HT patients, except for BMI and FT4. Previous studies show there are obvious elevated levels of TG, TGAb, TPOAb, and disordered TSH in patients with thyroid cancer (58–60), which were not analyzed in this study. Besides, poor prognostic clinical characteristics of thyroid cancer also include older age and large tumor size, which are usually detected in middle-aged women (39). However, these factors had no difference in our study.

Moreover, among the factors above, we found four factors that can be considered risk factors for HT development by adjusted logistic regression analysis: diabetes, UIC, FT3, and TSH. Finally, we utilized multiple factors to conduct machine learning modeling of HT. The verified risk factors inducing HT were analyzed in this study. Six predictive models were evaluated in the test set and the XGBoost model had the highest accuracy, precision, and AUC score. Also, LR, SVM, and MLP models also showed good performance. To date, there have been few studies on machine learning modeling of HT with multiple factors (32, 42). However, the machine learning modeling was not available to distinguish HT+C from HT with these factors. The arrangement of feature importance showed that 25-(OH)D was the best risk factor to indicate HT in comparison with the others. FT4 and TSH were the next two important factors of HT. We thereby suggest that deficient nutrition uptakes, including vitamin D and iodine, along with multiple factors related to thyroid hormones and lipid profiles, have great significance in the early diagnosis and risk prediction of HT. The usual diagnostic procedure of HT is long and it is difficult to confirm HT at early stage by ultrasound or cytological examinations. This study was the first time comprehensive factors of HT were taken into consideration. These factors made a superior contribution to the reliable performance of our models. From the perspective of the AUC score (0.781673), accuracy (0.729774), and precision (0.770717) of the XGBoost model, it is relatively valuable for the evaluation of HT risk and clinical decision. The AUC scores of LR, SVM, and MLP models are all 0.77, which indicate robust predictive performance of these multiple factors. The previous diagnosis models of HT were based on TPOAb and TGAb, which prompted AUC scores to 0.8 (32). However, since TPOAb and TGAb are the diagnostic criteria for HT, they were not suitable risk factors in our prediction modeling of HT. Moreover, the research may help identify novel preventative and therapeutic factors for HT patients and even in patients presenting with asymptomatic euthyroidism or subclinical hypothyroidism.

Nevertheless, there are still some further challenges to address. Because of the limited cohort study, a more clinical database in a larger cohort of HT patients should be collected to verify and improve the performance of the proposed models of machine diagnosis. In addition, for optimal machine learning modeling that distinguishes thyroid cancer from HT disease, other clinical characteristics and laboratory test data should be taken into account. Considering that HT is an autoimmune disease, lymphocytes and intestinal microbiota should be meaningful risk factors for clinical reference (33).

## Conclusions

In summary, we collected clinical information of HT patients and non-HT controls. Firstly, we found that the counts of thyroid goiter were significantly different among controls, patients with HT and thyroid cancer. Secondly, comprehensive factors including age, BMI, gender, diabetes, UIC, 25-(OH)D, FT3, FT4, TSH, TAG, TC, FPG, and LDL had significant correlations with HT, among which diabetes, UIC, FT3, and TSH were confirmed as important risk factors for HT development. Besides, XGBoost, LR, SVM and MLP models displayed appropriate predictive power in the machine learning modeling of HT. Among all the features, 25-(OH)D, FT4, and TSH played important roles in HT risk model construction. Our study firstly demonstrates that comprehensive examinations along with machine learning modeling can enhance the precision and efficiency of thyroid diagnosis, which may also enlighten better prevention and new therapeutic schemes for thyroid disorders.

## Data availability statement

The raw data supporting the conclusions of this article will be made available by the authors, without undue reservation.

## Ethics statement

The studies involving human participants were reviewed and approved by the Ethics Committee of Xuchang Central Hospital, Kaifeng Central Hospital and Jincheng People’s Hospital. Written informed consent to participate in this study was provided by the participants’ legal guardian/next of kin.

## Author contributions

PeL, FL, and MZ conceived the study, collected samples and wrote the manuscript. SX, PiL, JC, DT, YT, LZ, and XC performed laboratory experiments and collected data. YP analyzed data and established the machine learning models. HT provided guidance on data analysis and writing revision. YW was in charge of clinical database analysis and wrote the manuscript. YS edited and revised the manuscript. All the authors read and approved the final manuscript.

## Conflict of interest

Authors YP and HT were employed by company Shanghai Biotecan Pharmaceuticals Co., Ltd.

The remaining authors declare that the research was conducted in the absence of any commercial or financial relationships that could be construed as a potential conflict of interest.

## Publisher’s note

All claims expressed in this article are solely those of the authors and do not necessarily represent those of their affiliated organizations, or those of the publisher, the editors and the reviewers. Any product that may be evaluated in this article, or claim that may be made by its manufacturer, is not guaranteed or endorsed by the publisher.

## Supplementary material

Additional file 1 Thyroid ultrasound images of HT and PTC patients compared to healthy controls. **(A)** The normal thyroid ultrasound image of controls. **(B)** The thyroid ultrasound image of HT patients. **(C)** The thyroid ultrasound image of PTC patients.

Additional file 2 Specimens from healthy controls, patients with HT and PTC (HE staining, ×200). **(A)** Control shows normal thyroid follicular epithelial cells (EC), thyroid parafollicular cells (PC) and follicles filled with colloid (FC). **(B)** Typical changes of HT, including thyroid follicles with Hürthle-cell metaplasia (H), plasma cells (P) and lymphoid follicles with germinal centers (G). **(C)** Typical changes of PTC, such as papillary areas (PA) and follicular variant (FV).

Additional file 3 Machine learning modeling of thyroid cancer deteriorated from HT. The ROC curves of six models were shown with different colors. KNN, k-nearest neighbor classifier; LR, logistic regression; SVM, a support vector machine; DT, the decision tree model; MLP, the multilayer perceptron network; XGBoost, eXtreme Gradient Boosting.

## Abbreviations

HT, Hashimoto’s thyroiditis; PTC, papillary thyroid carcinoma; TIRADS, Thyroid Imaging Reporting and Data System; ICP-MS, inductively coupled plasma-mass spectrometry; ROC, receiver operating characteristic curve; AUC, area under curve; HT-C, HT patients without cancer; HT+C, HT patients with thyroid cancer; BMI, body mass index; UIC, urinary iodine concentrations; 25-(OH)D, 25 hydroxyvitamin D; FT3, free triiodothyronine; FT4, free thyroxine; TSH, thyroid stimulating hormone; TPOAb, thyroid peroxidase antibody; TAG, triglyceride; TC, total cholesterol; LDL-C, low density lipoprotein cholesterol; HDL-C, high density lipoprotein cholesterol; FPG, fasting plasma glucose; TGAb, thyroglobulin antibody; OR, odds ratio; AUC, area under curve; KNN, k-nearest neighbor classifier; LR, logistic regression; SVM, a support vector machine; DT, the decision tree model; MLP, the multilayer perceptron network; XGBoost, eXtreme Gradient Boosting.
