# Supplementary figures and images for: Prediction models constructed for Hashimoto’s thyroiditis risk based on clinical and laboratory factors
Source: Front Endocrinol (Lausanne). 2022 Aug 8;13:886953. doi: 10.3389/fendo.2022.886953 (PMC9393718; doi:10.3389/fendo.2022.886953)

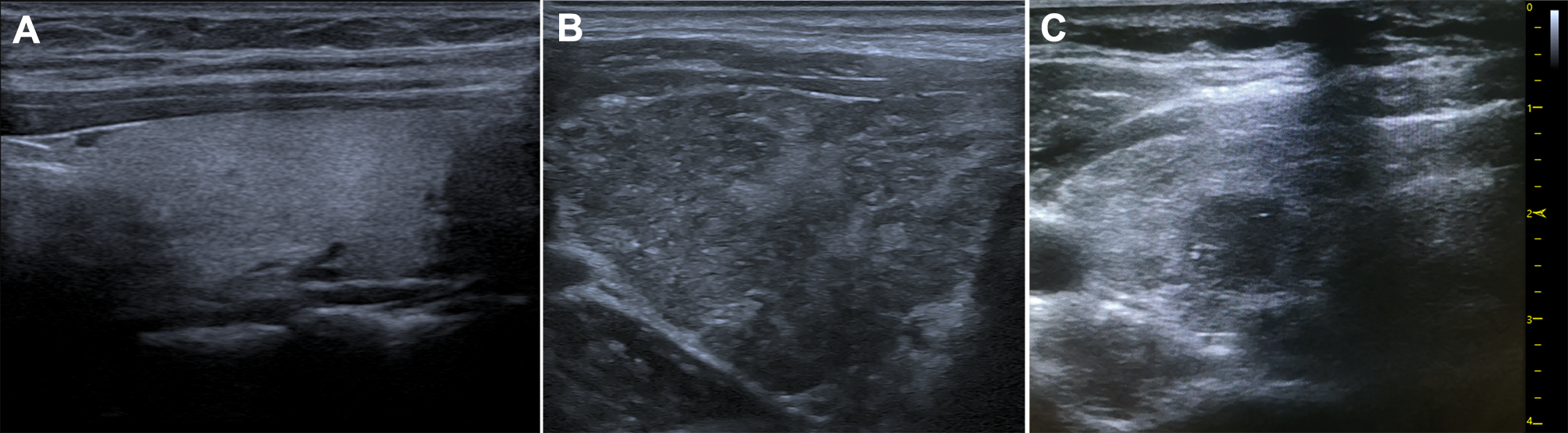

Supplement: Additional file 1 — Thyroid ultrasound images of HT and PTC patients compared to healthy controls. (A) The normal thyroid ultrasound image of controls. (B) The thyroid ultrasound image of HT patients. (C) The thyroid ultrasound image of PTC patients. [file Image_1.tif]

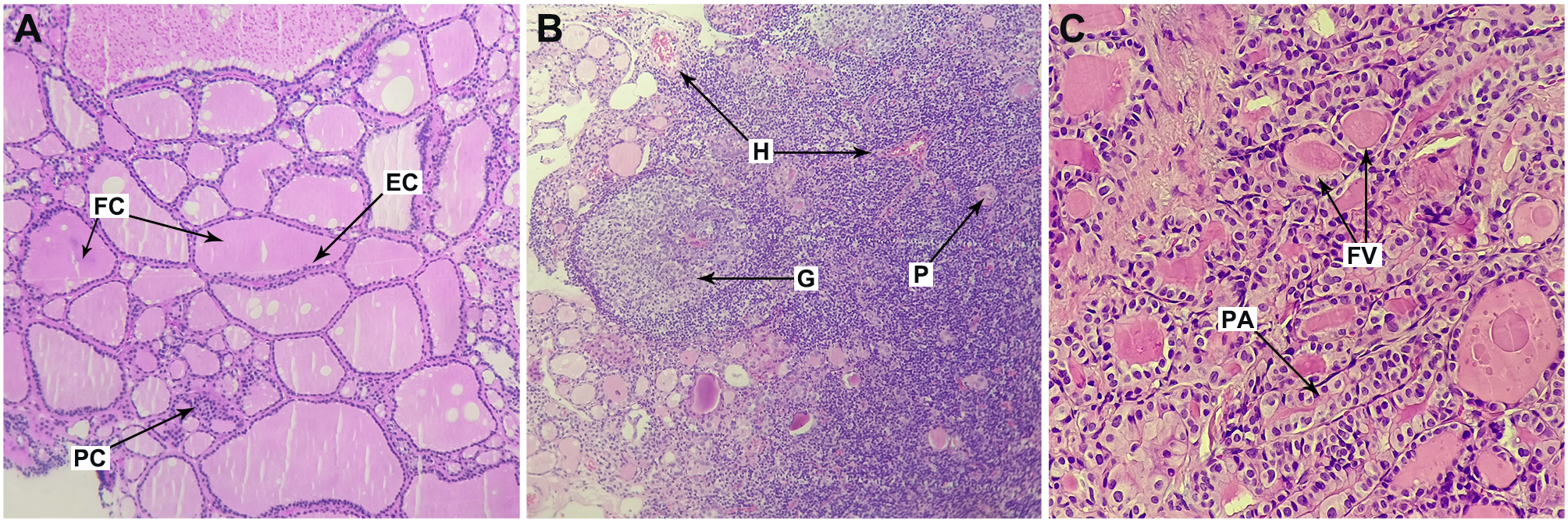

Supplement: Additional file 2 — Specimens from healthy controls, patients with HT and PTC (HE staining, ×200). (A) Control shows normal thyroid follicular epithelial cells (EC), thyroid parafollicular cells (PC) and follicles filled with colloid (FC). (B) Typical changes of HT, including thyroid follicles with Hürthle-cell metaplasia (H), plasma cells (P) and lymphoid follicles with germinal centers (G). (C) Typical changes of PTC, such as papillary areas (PA) and follicular variant (FV). [file Image_2.tif]

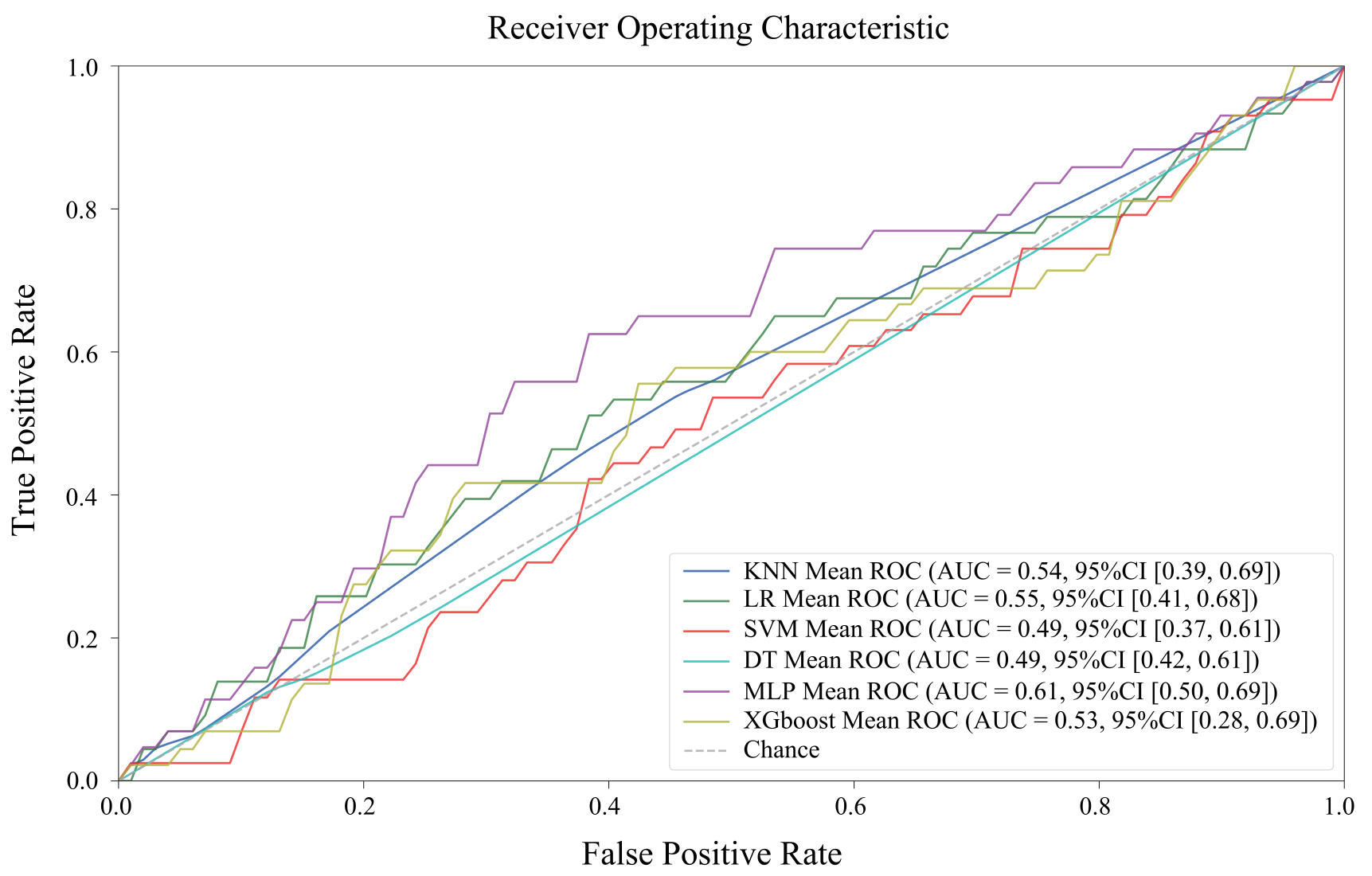

Supplement: Additional file 3 — Machine learning modeling of thyroid cancer deteriorated from HT. The ROC curves of six models were shown with different colors. KNN, k-nearest neighbor classifier; LR, logistic regression; SVM, a support vector machine; DT, the decision tree model; MLP, the multilayer perceptron network; XGBoost, eXtreme Gradient Boosting. [file Image_3.tif]
